# Supplementary material for: The Usefulness of a Duplex RT-qPCR during the Recent Yellow Fever Brazilian Epidemic: Surveillance of Vaccine Adverse Events, Epizootics and Vectors
Source: Pathogens. 2021 Jun 3;10(6):693. doi: 10.3390/pathogens10060693 (PMC8228867; doi:10.3390/pathogens10060693)
Supplement: Supplementary file 1 [file pathogens-10-00693-s001.zip › Supplementary Table.pdf]

Supplementary Table.:YFV strains isolated in six Latin American countries and in the USA over 40 years

Strain ID	Source Isolation	Specie	Year of isolation	Country	YFV genotype	Genbank
BEAN131	Monkey	Sentinel	1955	Brazil	SA-1	MW158338
BeAN23536	Monkey	*Cebus sp.*	1960	Brazil	SA-1	MW158339
BeAN142027	Monkey	*Saguinus midas*	1968	Brazil	SA-1	MW158340
BeAR233436	Mosquito	*Haemagogus sp.*	1973	Brazil	SA-1	MW158343
BeAR437159	Mosquito	*Haemagogus janthinomys*	1985	Brazil	SA-1	MW158344
BeAN510268	Monkey	*Alouatta sp.*	1991	Brazil	SA-1	MW158341
BeH844801	Human	*Homo sapiens*	2017	Brazil	SA-1	MF370531
BeH843297	Human	*Homo sapiens*	2017	Brazil	SA-1	MF370532
BeH843301	Human	*Homo sapiens*	2017	Brazil	SA-1	MF370533
OBS 5026	Human	*Homo sapiens*	1997	Equador	17DD	MW158362
OBS 2240	Human	*Homo sapiens*	1995	Peru	SA-2	MW158361
IQT 5591	Human	*Homo sapiens*	1998	Peru	SA-2	MW158359
IQD 8393	Human	*Homo sapiens*	2004	Peru	17DD	MW158358
MIS 1034	Human	*Homo sapiens*	2011	Peru	17DD	MW158360
CAREC889920	*Mosquito*	*Haemagogus janthinomys*	1988	Trinidad	SA-1	MW158345
CAREC890692	*Mosquito*	*Sabethes chloropterus*	1989	Trinidad	SA-1	MW158346
CAREC891954	*Monkey*	*Alouatta sp.*	1989	Trinidad	SA-1	MW158347
CAREC9515207	*Mosquito*	*Haemagogus sp*	1995	Trinidad	SA-1	MW158348
TR 7856	*Mosquito*	*Mansonia titillans*	2008	Trinidad	SA-1	MW158365
TR 7796	*Mosquito*	*Coquillettidia venezuelensis*	2009	Trinidad	SA-1	MW158364
TR 8183	*Mosquito*	*Culex (MeL) spissipes*	2009	Trinidad	SA-1	MW158366
TR 8194	*Mosquito*	*Coquillettidia venezuelensis*	2009	Trinidad	SA-1	MW158367
P 16065	*Human*	*Homo sapiens*	1966	USA	17DD	MW158363
INHRR 1A-04	*Monkey*	*Alouatta seniculus*	2004	Venezuela	SA-1	MW158349
INHRR 2A-04	*Monkey*	*Alouatta seniculus*	2004	Venezuela	SA-1	MW158350
INHRR 3A-05	*Monkey*	*Alouatta seniculus*	2005	Venezuela	SA-1	MW158351
INHRR 4A-05	*Monkey*	*Alouatta seniculus*	2005	Venezuela	SA-1	MW158352
INHRR 5A-05	Human	*Homo sapiens*	2005	Venezuela	SA-1	MW158353
INHRR 6A-05	Human	*Homo sapiens*	2005	Venezuela	SA-1	MW158354
INHRR 8A-06	*Monkey*	*Alouatta seniculus*	2006	Venezuela	SA-1	MW158355
INHRR 10A-10	*Monkey*	*Alouatta seniculus*	2010	Venezuela	SA-1	MW158357

Legend: SA-I = Southamerica genotype I; SA-II = Southamerica genotype II
