# Supplementary figures and images for: The Usefulness of a Duplex RT-qPCR during the Recent Yellow Fever Brazilian Epidemic: Surveillance of Vaccine Adverse Events, Epizootics and Vectors
Source: Pathogens. 2021 Jun 3;10(6):693. doi: 10.3390/pathogens10060693 (PMC8228867; doi:10.3390/pathogens10060693)

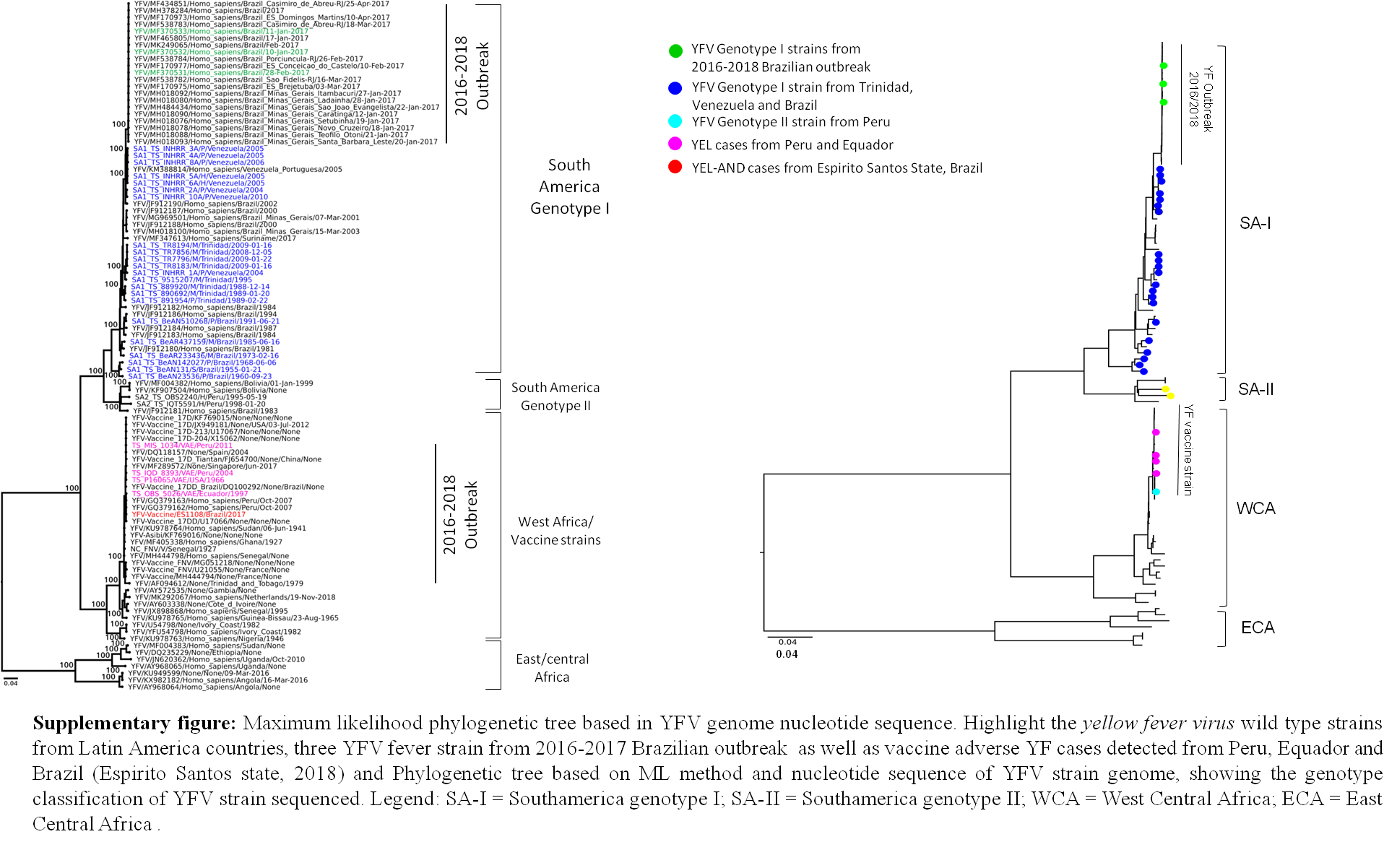

Supplement: Supplementary file 1 [file pathogens-10-00693-s001.zip › Supplementary Figure.png]
